# Ice Water in Cholera Infantum

**Published:** 1890-11

**Authors:** E. McD. Bridgeford

**Affiliations:** 1751 N. 11th street; St. Louis


For Daniel's Texas Medical Journal.

### ICE WATER IN CHOLERA INFANTUM.

BY E. MCD. BRIDGEFORD, M. D., ST. LOUIS.

OF ALL diseases most dreaded by the parents of "our dear little ones" cholera infantum stands at the head of the list. The extreme heat that we have had to endure since June 1st has certainly been a great factor in causing the death of hundreds of children in this city. In the overcrowded tenement houses the mortality has been very high. Good hygiene in such a place as the Ashley building is impossible, and certainly the physician is almost powerless under such circumstances to do any good. I do not claim to be the originator of the ice water treatment for the disease under consideration, but wish to give my experience with it, and also my method of treating cholera infantum.

I suppose it would be proper in this place to state something of the history of this mode of treatment. My attention was first attracted to the use of ice water in cholera infantum by reading an article, which had been read before the St. Louis Medical Society by Dr. H. F. Hendrix, of this city. This gentleman's views are, I think, very rational. He claims "that by the watery discharges of cholera infantum the blood and tissues of the body are robbed of their liquid constituents and this fluid must be replaced or in a short time the blood will become so depleted that it can no longer furnish nutrition to the vital organs." It has been the practice of mothers for ages to give their babies water "that has had the air taken off." This way of taking the air off is various—it is not necessary to explain their process. However we do know that such water is not a very cooling or delicious drink and is in almost every instance vomited about as soon as it reaches the stomach. With ice water such is not the case—it is nearly always retained by the stomach for five minutes or more, and then, if it is rejected by that organ, some of the water has been absorbed and goes into the blood current, freighted with oxygen, which gives life to the powers that are rapidly famishing. I believe Dr. Hendrix gives a little bicarbonate of soda in each glass of water. Now I come to my method of treating a case. I do not, as will soon be seen, depend entirely on the ice water. As soon as I see the case and make a diagnosis of cholera infantum I order a glass of *ice* water and allow the child to drink *all* it wants,—if this is vomited in one or two minutes, I order a second glass which is likely to be retained. If the hands and feet are cold (and this is nearly always the case) I direct a warm bath to be given to the trunk and extremities and a cool cloth to be placed on the head. After this has been attended to, I prescribe the following—supposing the child to be ten or fourteen months old:

℞ $\frac{1}{10}$ triburates hydrarg chlor mite No. 10.
Sig. 1 triturate every 3 hours.

℞ Bismuth sub. nit. gr. xxiv,
Mistura cretæ (U. S. D.) ℥ iss,
Acidi carbolici, gtt. iii,
Aquæ calcis, ℥ ss,
Aquæ mentha piper ad. ℥ iii.
M. Sig. A teaspoonful every two hours.

I usually give the last prescription until four doses have been taken, and then give the same size dose every three or four hours.

I most always find that the watery discharges become thicker in a few hours, are of a neutral reaction, while at the onset of the attack the *reaction* is *acid*. I never use *opium in any form*. My success with this line of treatment has been very flattering, and I hope, kind reader, if you have not used ice water in the treatment of cholera infantum, you will give it a trial, and I am sure you will save more of the little ones, and be delighted with the result. Let us hear from you. 1751 N. 11th street.

